# External post-mortem examination in virtual reality—scalability of a monocentric application

**DOI:** 10.1007/s00414-024-03229-9

**Published:** 2024-04-09

**Authors:** Christina Klus, Katja Krumm, Sindy Jacobi, Marie-Christin Willemer, Charlotte Daub, Dietrich Stoevesandt, Katrin Metzler, Carolin Richter, Lisa-Maria Peter, Steffen Heide, Uwe Schmidt

**Affiliations:** 1 Faculty of Medicine, Martin-Luther-University Halle-Wittenberg, Dorothea-Erxleben-Lernzentrum-Halle (DELH), Magdeburger Straße 12 (Saale), 06112 Halle, Germany; 2 Carl Gustav Carus Faculty of Medicine, TUD Dresden University of Technology, Institute of Medical Education, Medical Interprofessional Training Centre (MITZ), Dresden, Germany; 3 Carl Gustav Carus Faculty of Medicine, TUD Dresden University of Technology, Institute of Forensic Medicine, Dresden, Germany; 4 Institute for Forensic Medicine, Martin Luther University of Halle-Wittenberg, Franzosenweg 1 (Saale), 06112 Halle, Germany

**Keywords:** Forensic medicine, Virtual reality, Serious game, Medical education, Computer-based learning, Digitalised teaching

## Abstract

Conducting external post-mortem examinations is an essential skill required of physicians in various countries, regardless of their specialization. However, the quality of these examinations has been a subject of continuous debates, and notable errors were reviled. In response to these shortcomings, a virtual reality (VR) application was developed at Halle's medical department in Germany, focusing on the scene of discovery and the completion of death certificates. The initial trial of this VR application in 2020 involved 39 students and 15 early-career professionals. Based on the feedback, the application underwent improvements and was subsequently introduced to the medical department in Dresden, Germany, in 2022. Its primary objective was to showcase the VR training's adaptability and scalability across various educational structures and levels of medical expertise. Out of 73 students who participated, 63 completed the evaluation process. 93.1% (n = 58) of the evaluators reported increased confidence in conducting external post-mortem examinations, and 96.8% (n = 61) felt more assured in filling out death certificates, crediting this progress to the VR training. Additionally, 98.4% (n = 62) believed that repeating forensic medical aspects in their coursework was crucial, and 96.8% (n = 61) viewed the VR examination as a valuable addition to their academic program. Despite these positive responses, 91.6% (n = 55) of participants maintained that training with real corpses remains irreplaceable due to the insufficiency of haptic feedback in VR. Nevertheless, the potential for enhancing the VR content and expanding the training to additional locations or related disciplines warrants further exploration.

## Introduction

In several countries, including Germany, physicians are required to perform external post-mortem examinations regardless of their specialization [1, 2]. The quality of these medical examinations is often intensively and sometimes controversially discussed, particularly due to structural conditions and occasionally glaring faults. [3–7]. One potential solution to this issue could be to intensify the training of medical students with a stronger practical focus. [8, 9] However, a review of the current situation in forensic medical teaching reveals that, despite several published reports on new teaching, learning, and examination methods regarding medical external post-mortem examinations [9–14], conventional formats still prevail in Germany [15, 16]. Therefore, the challenge remains to foster the establishment of practice-oriented teaching and learning methods, develop corresponding exam formats and distribute these concepts to further sites.

At the Institute of Forensic Medicine, a faculty of Martin Luther University Halle-Wittenberg's medical department (Germany), the topic of external post-mortem examination comprises of a lecture (90 min) and practical exercises (180 min). Until 2014 students in their 8th semester acquired practical skills exclusively on deceased individuals during a seminar in a mortuary. To improve the medium- and long-term quality of the examinations as well as to enlarge the practical training a cooperation with the Dorothea Erxleben Learning Center Halle was found. Initially, a skills lab station and an e-learning module were established, where students could practice filling out death certificates in 10 different external post-mortem examination scenarios [17]. Corresponding to this training and the practical exercises with real corpses, two forensic medicine OSCE stations (Objective Structured Clinical Examination) were developed. These included the practical training of an external post-mortem examination on a simulation mannequin and the completion of a death certificate in the presence of an examiner. Shortly afterwards the second station was transferred to a computer-based format [18]. Another purpose of the simulation mannequin modified for external post-mortem examination was to test the corresponding practical skills for the medical final examination during the COVID-19 pandemic [19]. In a first collaboration with the forensic medicine institute and the Medical Interprofessional Training Center (MITZ) of the Technical University of Dresden (TUD), the simulation mannequins were also utilized for training of an external post-mortem examination for medical students at MITZ [20]. The scenario was supplemented by actors simulating the relatives of the deceased. Furthermore, in Dresden, this method was also applied to a training and assessment of external post-mortem examination for police officers, opening up a new field of application. However, a critical review of the applied methods revealed that the comprehensive setting at the scene of the body's discovery could not always be adequately considered or simulated. One way to address this problem is the reconstruction of real cases using model rooms. As these environments require additional preparations and personnel expenditure cost-effective alternatives had to be determined. It was noted that especially clinical disciplines have already introduced new digital teaching and learning environments for several years, particularly using virtual reality (VR) technology. Moreover, virtual environments are said to have resource-saving and thus cost-efficient characteristics, which can simulate real scenarios in a safe environment [21].

With regard to this development a virtual external post-mortem examination project was started in 2018 based on a close collaboration between the Institute of Forensic Medicine Halle and the Dorothea Erxleben Learning Center Halle. The virtual reality application was created in Unity Engine (Unity Technologies, San Francisco, CA, USA, deployed for Oculus Quest VR glasses, Menlo Park, CA, USA) and the main objective was to provide a more realistic and detailed discovery site of a corpse [22, 23]. From there on the virtual body examination scenario was subjected to an initial evaluation with a limited number of teachers and medical students in their practical year [22]. Subsequently, a further case scenario was developed, along with additional modifications to the process and technical implementation. Students were trained with two virtual cases. As the simulation focuses on typical circumstances of discovery the training is based in a domestic environment. Each scenario comprises of a deceased person with post-mortem signs describing a specific cause of death. Players are expected to perform a body examination by using an integrated toolbar which allows for the complete undressing of the corpse using scissors, measuring the core body temperature with a thermometer, examining eyes and mouth with a pair of tweezers and a closer inspection of livor mortis or other external abnormalities using a magnifying glass function. In addition to this, players have the opportunity to investigate the virtual apartment and the belongings of the inhabitant. Further hints such as an identity card for identification purposes, provide necessary information for processing the case. Afterwards students complete a death certificate in the virtual environment. The seminar is concluded with a final discussion and feedback from a tutor [23]. Further evaluations were then carried out with students in their practical year and doctors in advanced training [23]. In light of the need for efficient and widespread resource use and the exploitation of synergistic potentials, interprofessional and interdisciplinary teaching concepts should be transferred across locations [24, 25]. Therefore, the question arose as to whether, the successful establishment at the Halle site could be repeated at other locations. Consequently, in 2022, the VR external post-mortem examination was modified in Halle and the didactic concept was altered and implemented at the MITZ Dresden as part of the virTUos-project at TUD funded by “Stiftung Innovation in der Hochschullehre”.

## Material and methods

In order to pursue a successful transfer of the application to the curricula of MITZ which is part of the medical faculty Dresden, a comprehensive modification for integration into the existing training concept of forensic medicine and the local conditions of MITZ was necessary. At the Dresden location, thanatology has so far been covered in a lecture (90 min) and a practical course on external post-mortem examination (90 min) in seminar group size (about 20 students) in the 5th semester, plus the opportunity for training in medical corpse examination using a simulation mannequin (45 min) in the 8th semester. Furthermore, the optional elective "Medicine and Law" is provided during the 2nd semester, which also includes a practical exercise on external post-mortem examination. During the winter semester of 2022/23, the virtual external post-mortem examination was offered as an elective course in a peer-teaching format for students in their 5th semester, for the first time. In that respect it was advantageous that a system update to SteamVR in 2022 enabled the serious game to be available across device manufacturers and plattforms. SteamVR is a free plugin provided by Valve Corporation on the video game distribution service Steam and offers a plug-and-play solution for SteamVR-compatible devices, including the HTC Vive and Meta Quest 3 and Pico Neo 4. As part of the alterations for the new Dresden site, the user interface was also fundamentally revised. While the possible functions remained the same as those in Halle, the users were now guided step by step through the process of filling out the death certificate. Information collected in the game appeared in the smart menu and could be linked with the corresponding sections of the death certificate. In contrast to the first evaluation phase in Halle, in which the course was compulsory and only took place on site, the optional course in Dresden was offered using the "flipped classroom" method and consisted of two phases. As preparation for the event an e-learning module was tailored, comprising a summary of the required theoretical learning content, previously imparted during lectures and practical training, as well as a checklist for filling out a death certificate and practical repetition exercises.

During the on-site training, each group comprised of four students. At the beginning of the one-hour event, trained tutors introduced the topic and explained the technical conditions. While the students took turns performing the virtual external post-mortem examination, the progress was documented by the other participants using a checklist. This was followed by a work phase and a reflection phase. The experiences and insights gained in the virtual environment were recapitulated and then transferred to the death certificates.

The pilot stage was evaluated using a uniform, standardized evaluation form. Based on 19 items previously used at the Halle site [22] the evaluation was expanded to 31 items and categorized into six areas: prior knowledge (4 items), organization and structure (6 items), learning content (5 items), guidance by the tutors (4 items), technical implementation of Virtual Reality (6 items), and overall assessment (6 items). 22 items were rated using a Likert scale. Level 1 indicated full agreement, while the highest level 5 symbolized fundamental disagreement. On the 5-point scale, levels 1 and 2 were summarized as 'full and predominant agreement', as previously in Halle [22, 23]. For two items, specific details (semester of study, previously completed courses) were requested, while for the other six items, respondents usually had three response categories available: 'just right', 'too little', and 'too much'. Finally, a free text field allowed participants to state particularly critical or positive aspects.

## Results

A total of 73 students participated in the pilot stage, 63 of whom completed an evaluation form (response rate of 86%). However, not all of the participants rated each item, which is indicated in the following by the varying total number of evaluations. The participants (where specified) predominantly belonged to the 7th semester (n = 20), followed by the 8th semester (n = 17), 9th semester (n = 15), and a few from the 6th (n = 3) and 5th semester (n = 4).

**Fig. 1:**
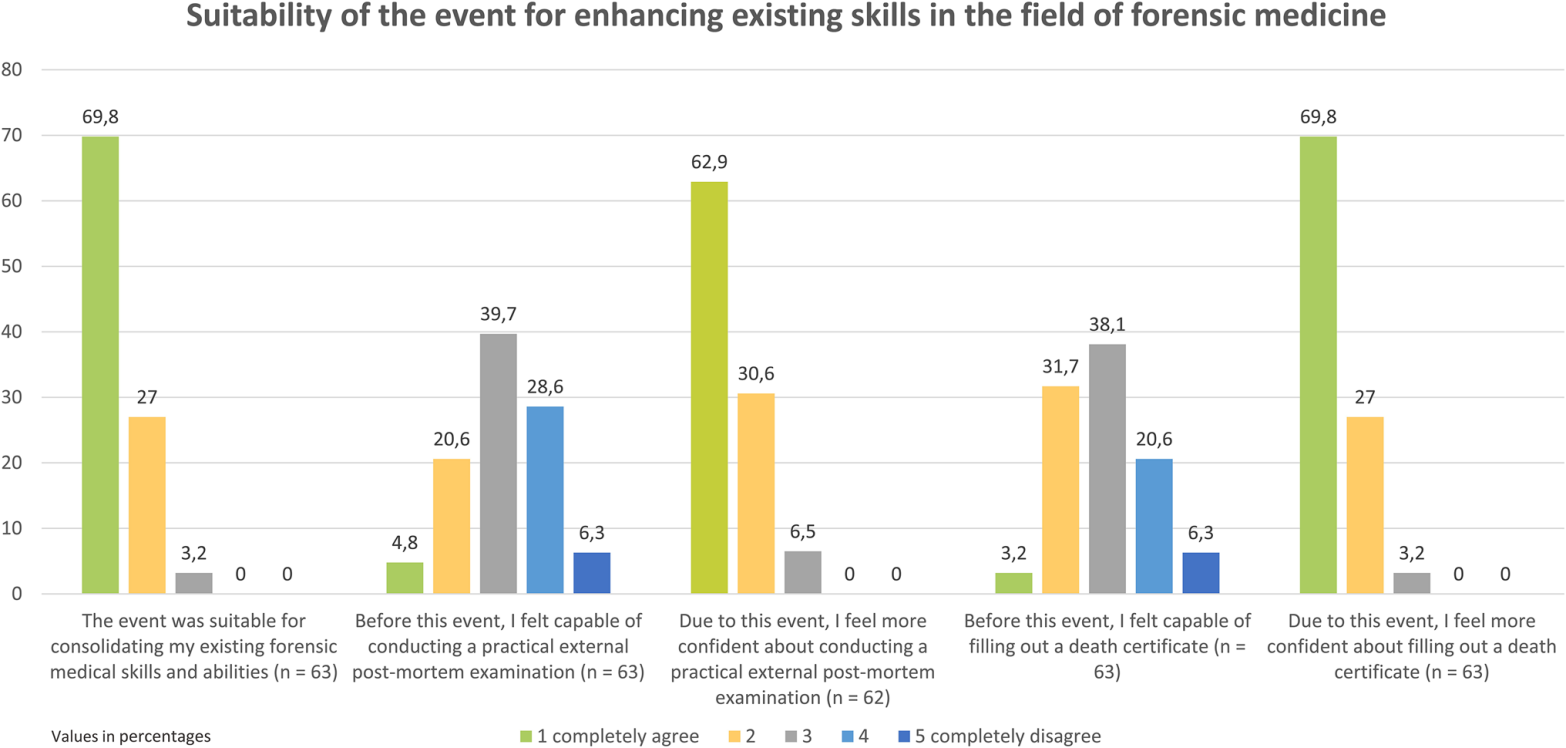
Suitability of the event for enhancing existing skills in the field of forensic medicine

At the time of the event, most students already had prior knowledge of thanatology. 58 participants (85.7%) had attended the lecture, and 43 students (68.3%) had completed the practical course in corpse examination. Despite this background, only 25.4% of the participants (n = 16) felt completely or mostly confident in conducting a practical corpse examination before the learning event (Fig. 1). As for completing a death certificate, this percentage was 34.9% (n = 22).

The e-learning module provided for preparing for the virtual external post-mortem examination was assessed as completely or mostly positive in both scope (95.1%, n = 58 of 61) and content (93.1%, n = 54 of 58) by the majority of participants. Only four participants reported technical issues during the module. Approximately four-fifths (87.3%, n = 55) of the students found the information about the procedure of the actual teaching event to be completely or mostly sufficient.

All participants deemed the simulation of the external post-mortem examination in small groups of four students each to be optimal. The one-hour duration of the teaching event was considered too short by just over a quarter of the participants (28.6%, n = 18), while a significant majority (69.8%, n = 44) found the timing to be appropriate. The timeframe was regarded as rather too long by only one participant. Both the technical (93.4%, n = 57 of 61) and subject-specific instruction (96.8%, n = 61) provided by the tutors were rated as just right. Almost all students (98.4%, n = 62) felt encouraged to think independently and were appropriately challenged by the tutors according to their knowledge level.

Approximately three-quarters of the students (79%, n = 49 of 62) had no or only minimal prior experience with VR technology. As a result, only half of the participants (53.3%, n = 32 of 60) reported that they found the practical operation of the VR headset to be easy or very easy. Among the participants with previous experience in VR, just under half reported having suffered from nausea and dizziness ("motion sickness") at least occasionally during use (48.3%, n = 14 of 29). A slight majority of the students (53.3%, n = 32 of 60) considered the technical implementation of the scenario to be realistic.

Due to the course, 93.1% (n = 58 of 62) felt more confident in conducting a practical post mortem examination, an increase from the initial rate of 25.4%. (Fig. 1) When it came to filling out the death certificate, there was a clear improvement from 34.9% to 96.8% (n = 61 of 63) in feeling completely or mostly competent (Fig. 1).

**Fig. 2:**
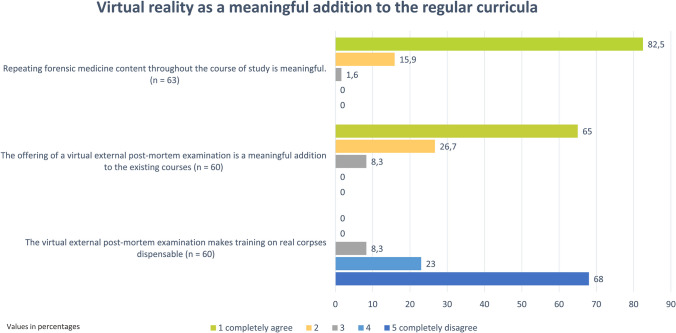
Virtual reality as a meaningful addition to the regular curricula

Nearly all students (98.4%, n = 62 of 63) completely or mostly agreed that aspects of forensic medicine should be repeated throughout their studies (Fig. 2). Similarly, 96.8% (n = 61 of 63) believed that simulated external post-mortem examination using VR technology were completely or mostly suitable for reinforcing their forensic skills and competencies. Additionally, the virtual external post-mortem examination was seen as a useful complement to existing teaching events by 91.7% (n = 55 of 60) of the students (Fig. 2). The technical implementation of the teaching event was judged as mostly or completely successful by 91.8% (n = 56 of 61), and the content approval rate was even higher at 96.8% (n = 61 of 63). The teaching event received a top grade of "1" from 68.3% (n = 43 of 63) of the participants, 28.6% (n = 18 of 63) rated it as "2", and 3.2% (n = 2 of 63) as "3". Finally, 96.9% (n = 61 of 63) of the students indicated they would very likely or likely recommend the course to others. Despite the high approval rates for the newly introduced learning concept, 91.6% (n = 55 of 60) of the participants expressed that training on a real corpse is still indispensable (Fig. 2).

## Discussion

The issue of errors in external post-mortem examinations, extending to missed homicides, is exceedingly complex [3, 4, 26, 27]. Several aspects can classify the causes of these errors: inherent limitations of the external examination itself, structural factors such as legal regulations, situational factors like family expectations for a natural death determination or challenging conditions such as decomposition, police biases favoring natural death cases, and medical reasons [5, 26]. These medical errors encompass a broad range, from incomplete examinations without inspection of body orifices to overlooking subtle or evident signs of external violence, careless identification of the body, misjudgments regarding the time of death, cause of death, manner of death, and the incorrect formation and classification of causal chains. [2, 5, 26, 28]. From the perspective of forensic medicine, an important contribution to reducing this issue can be made by providing more intensive and practice-oriented training for medical students, as well as continuing education for doctors obliged to conduct external post-mortem examinations. In recent years, several concepts for corresponding teaching, learning, and examination methods have been introduced, but they have not yet become widespread. Schmeling et al. [10] described a web-based e-learning program in the form of a "Click and Point Adventure" for training in external post-mortem examination, which also takes into account the environment of the corpse. Ebert et al. [29], replicated a crime scene by reconstructing it in virtual reality, while Koller et al. [30] used the VR environment for the specific tasks of external forensic examination and measurement of injuries. Another analog variation is the already described training with simulation mannequins and the additional participation of actors as simulation patients [20], which also represents a comprehensive setting for corpse inspection. In all mentioned scenarios, practical training for external post-mortem examination is emphasized, and the teaching of how to fill out a death certificate is omitted. Also current practical exams reflect this strategy, where the actual performance of the external post-mortem examination and the filling out of the death certificate are separated. Especially during exams, such as OSCE exams (Objective Structured Clinical Examination), the tight time schedules are apparent which lead to considering the corpse environment only marginally [18]. This assessment is likely to apply equally to the integration of this topic into OSPE formats (Objective Structured Practical Examination) [11, 12].

In order to largely cover the spectrum of potential medical sources of error, both the practical external post-mortem examination with consideration of the environment and the completion of the death certificate in a case scenario are intertwined in the virtual external post-mortem examination [22]. Throughout the first evaluation phase at the Halle site, the differences and potential advantages over previous projects were demonstrated. In the current study, alongside an updated inventory of similar projects and a comparison to these applications, the main focus was to examine to what extent such a learning concept can also be successfully disseminated across different locations. Likewise, the limitations and boundaries of the method must also be critically discussed.

Before the virtual external post-mortem examination, almost all students had attended the lecture on thanatology, and over two-thirds had completed the external post-mortem examination practicum. As a result of these courses, students generally assessed their own skills and competencies in conducting practical external post-mortem examinations and filling out death certificates rather conservatively, as it had been observed previously at the Halle site [22]. This assessment supports the need to establish additional teaching and learning methods for the medical external post-mortem examination that goes beyond the current, still widely used concept of lectures and practicums [15].

Unlike the approach at Halle, an e-learning module was introduced in Dresden as a preparatory tool for the virtual external post-mortem examination and included additional items in the evaluation. Both the self-study unit for preparation and the materials provided were met with a high proportion of favorable evaluations. These results suggest that the modification of the onboarding phase implemented in Dresden should also be adopted in Halle and possibly at other locations. At this point, the modular usability of the virtual reality application also becomes apparent. Acting as a practical component within forensic medicine educational offerings, it was able to supplement dissemination of knowledge by a tutor (Halle) as well as an e-learning programme (Dresden). Additional fields of application are conceivable and should be investigated.

The duration of one hour deemed appropriate by nearly two-thirds of the students, and the group size of four participants, which was rated as optimal by all attendees, suggest that the time frame of at least one hour and the small group format should be maintained in the future. This is especially pertinent since teaching units conducted in small groups are associated with higher learning success compared to larger group formats [31, 32]. Simultaneously, considering the preferences of students who favor a longer format and the evaluation results of the pilot study [22], it would be advisable to extend the course offering to two hours, provided the time frame allows it.

Both the technical and the subject-specific support provided by the tutors was rated as exceptionally helpful, yet there was still ample space left for encouraging independent thinking. This balance was also positively highlighted by the students in their open-ended feedback, referring to it as a "pleasant, educational atmosphere." During the course, the support from the tutors, including the possibility of intervention, seems justified given that for most participants, handling virtual reality was largely unfamiliar. In fact, three quarter of students stated no or little experience with VR applications in Dresden which was a consistently high rate and similar to the initial assessment in Halle, four years earlier [22].

Correspondingly, the assessment of the practical operation of the VR format was balanced between easy and difficult handling. According to Speidel et al. [33], student reservations are due to a lack of experience with VR technology, and its significance for current and future teaching is viewed with some skepticism. Although it is expected that such concerns will decrease as digitalization in the work and leisure environment continues to grow, especially accelerated by the COVID-19 pandemic, this challenge must still be adequately considered when further establishing VR technology in medical education, along with the well-known issue of "motion sickness." In Dresden, this aspect was included as an additional item in the evaluation, with over 40% of participants indicating that they had faced this phenomenon at least occasionally during previous VR usage. As motion sickness is a known issue with VR applications in other fields, a workaround was implemented through task distribution in small groups. This allowed students to follow activities by "looking over the shoulder," eliminating the need to wear VR goggles for the entire duration of the course.

In the 2020 study in Halle, the technical implementation and realism of the simulated external post-mortem examination using VR technology were rated as fully or predominantly positive by almost two-thirds of the participants. In the meantime, the application has been further developed and optimized. However, the realism was rated positively by only 53.3% of the participants in Dresden, whereas the technical implementation of the virtual external post-mortem examination was rated as fully or predominantly positive by 91.8%. Since primarily modifications have been made to the interaction concept and the spatial design has remained largely unchanged, an increased demand for aesthetics in virtual environments can be inferred, which may have arisen due to advancements and expectations from the gaming industry. To continuously ensure a realistic representation, it is necessary to optimize the environment according to current standards, now, that this increased expectation has been detected.

Regarding the content and learning objectives of the VR training, the evaluation results showed that, at least according to the participants' assessments, forensic skills and abilities could be significantly enhanced. Over 90% of the participants felt much more confident in conducting a practical external post-mortem examination as well as filling out a death certificate after the course. These approval rates were significantly higher than in the previous study in Halle [22], where the acceptance rate for the practical external post-mortem examination was at 52.5% and for filling out the death certificate was at 62.5%. Whether these differences can be solely attributed to the modified concept in Dresden with an online preparation unit and the technical optimization of the VR application is questionable and should be examined in more detail. In the same way the extent of the actual long-term learning success should be assessed in a corresponding examination format. Furthermore, the course should also be evaluated in a larger cohort than in the limited number of participants in the present pilot event.

In the overall assessment, nearly all participants (98.4%) deemed the repetition of forensic medical content as meaningful, and the virtual external post-mortem examination was predominantly positive (87.1%) considered a suitable didactic tool for this purpose. These approval rates were even slightly higher than those in the Halle study [22], which showed 85.0% for meaningful repetition of content and 64.1% for the suitability of the virtual external post-mortem examination as a didactic instrument. Moreover the highly positive reception of the newly introduced learning concept among students in Dresden was reflected in the overall grades and the rate of recommendation. Other studies have also attributed high didactic potential to VR technology due to its more sustainable learning effects compared to other digital and traditional teaching methods [33, 34].

In the applications in Dresden and Halle, it was determined that the simulated external post-mortem examination using VR technology offers advantages over the real post-mortem examination. These include higher standardization and reproducibility of findings, as well as the effectiveness and independence from spatial, temporal and situational resources [22]. Based on these advantages, the use of VR technology as a testing method is recommended, especially since simulation mannequins have already been successfully used in the practical examination of external post-mortem examination [18, 19]. Whereas, in accordance with Speidel et al. [33], the reliable establishment of teaching formats using VR technology is a prerequisite.

However, in terms of sensory feedback, the simulated external post-mortem examination using VR technology has multiple inherent weaknesses, which comprise the absence of tactile and olfactory sensory impressions and the consequent lack of need to overcome emotional barriers [22], which are important causes of errors in real external post-mortem examinations [26]. Based on these experiences from the 2020 evaluation, the issue was addressed in Dresden as well, confirming that for over 90% of the students, training on a real corpse remains indispensable despite the learning effect of the virtual external post-mortem examination. Therefore, to achieve medium and long-term improvement in the quality of medical post-mortem examinations, pursuing intensified practical training is recommended, involving a combination of training on both real and virtually simulated corpses.

## Conclusion and future directions

Overall, it can be concluded that the simulated external post-mortem examination using VR technology offers numerous advantages and high didactic potential, along few disadvantages. Undoubtedly, significant technical, material, and personnel efforts were required for the establishment [22], further development [23] as well as functioning cooperation structures between the involved institutions and a high commitment of the participating individuals. At the same time, the present study has shown that the simulated external post-mortem examination using VR technology can be successfully transferred to other locations and adapted to local conditions. For the efficient application and further development of this learning method, it would be desirable for more sites to participate. After agreeing on appropriate transfer conditions [25], a larger contingent of various cases could be established for exchange between locations. Furthermore, a type of modular system of suitable learning concepts could be developed, which would address specific curricular requirements. The content of the modules as well as the combination and sequence of the learning content and the impact on learning outcomes depending on prior knowledge must be scrutinized to derive recommendations for action. In order to record comparable user experiences these measures can be accompanied by the automated documentation of user decisions, as well as conducting comparable user tests or establishing suitable test formats. Beside this cross-location dissemination and documentation of increased knowledge, the use of the simulated external post-mortem examination is also conceivable beyond its application in medical studies. Especially in countries with a medical obligation to perform external post-mortem examinations regardless of specialization, this learning method can be applied in the continuing education of physicians. Subsequently, the virtual external post-mortem examination could be implemented in training measures for other professional groups, such as the police or interprofessional fields such as criminology.

## Data Availability

The authors confirm that all data generated or analysed during this study are included in this published article.

## References

[1] Das C (2005) Death certificates in Germany, England, The Netherlands, Belgium and the USA. Eur J Health Law 12:193–211 17302044 10.1163/157180905774857916

[2] Schröder AS, Wilmes S, Sehner S, Ehrhardt M, Kaduszkiewicz H, Anders S (2017) Post-mortem external examination: competence, education and accuracy of general practitioners in a metropolitan area. Int J Legal Med 131(6):1701–1706 28210814 10.1007/s00414-017-1559-9

[3] Brinkmann B, Banaschak S, Bratzke H, Cremer U, Drese G, Erfurt C, Giebe W, Lang C, Lange E, Peschel O, Philipp KP, Püschel K, Riße M, Tutsch-Bauer E, Vock R, Du Chesne A (1997) Fehlleistungen bei der Leichenschau in der Bundesrepublik Deutschland. Arch Kriminol 199:65–74 9157831

[4] Karger B, Lorin de la Grandmaison G, Bajanowski T, Brinkmann B (2004) Analysis of 155 consecutive forensic exhumations with emphasis on undetected homicides. Int J Legal Med 118:90–94 14986016 10.1007/s00414-003-0426-z

[5] Zack F, Kaden A, Riepenhausen S, Rentsch D, Kegler R, Büttner A (2017) Fehler bei der Ausstellung der Todesbescheinigung. Rechtsmedizin 27:516–527 10.1007/s00194-017-0193-7

[6] Lahti RA, Penttilä A (2001) The validity of death certificates: routine validation of death certification and its effects on mortality statistics. Forensic Sci Int 115(1–2):15–32 11056267 10.1016/S0379-0738(00)00300-5

[7] Winkel BG, Holst AG, Theilade J et al (2012) Differences in investigations of sudden unexpected deaths in young people in a nationwide setting. Int J Legal Med 126:223–229 21779923 10.1007/s00414-011-0602-5

[8] Rothschild M (2009) Probleme bei der ärztlichen Leichenschau. Rechtsmedizin 19:407–412 10.1007/s00194-009-0627-y

[9] Heide S, Lessig R, Hachmann V, Stiller D, Rönsch M, Stoevesandt D, Biolik A, Watzke S, Kellner J (2018) Establishment of two forensic medicine OSCE stations on the subject of external post-mortem examination. Int J Legal Med 132:311–319 28634679 10.1007/s00414-017-1630-6

[10] Schmeling A, Kellinghaus M, Becker JC, Schulz R, Schäfer A, Pfeiffer H (2011) A web-based e-learning programme for training external post-mortem examination in curricular medical education. Int J Legal Med 125(6):857–861 21901359 10.1007/s00414-011-0613-2

[11] Pramod Kumar GN, Sentitoshi ND, Menezes RG, Kanchan T (2015) Student’s perspectives on objective structured practical examination (OSPE) in forensic medicine—a report from India. J Forensic Legal Med 32:39–41 10.1016/j.jflm.2015.02.013 25882148

[12] Menezes RG, Nayak VC, Binu VS, Kanchan T, Rao PP, Baral P, Lobo SW (2011) Objective structured practical examination (OSPE) in forensic medicine: students’ point of view. J Forensic Legal Med 18(8):347–349 10.1016/j.jflm.2011.06.011 22018165

[13] Anders S, Mueller M, Sperhake JP, Petersen-Ewert C, Schiekirka S, Raupach T (2014) External post-mortem examination in undergraduate medical education—what do students really learn? Int J Legal Med 128(6):1031–1038 24487723 10.1007/s00414-014-0974-4

[14] Martínez-Jarreta B, Monsó E, Gascón S, Casalod Y, Abecia E, Kolb S, Reichert J, Radon K (2009) NetWoRM team. E-learning strategies in occupational legal medicine based on problem solving through “CASUS” system. Leg Med 11(Suppl 1):313–314 10.1016/j.legalmed.2009.02.075 19362874

[15] Nold S, Anders S, Bajanowski T, Heide S (2020) Inhaltliche und strukturelle Änderungen der rechtsmedizinischen Lehre in Deutschland. Rechtsmedizin 30:225–232 10.1007/s00194-020-00397-x

[16] Nold S, Heide S, Bajanowski T, Anders S (2021) Studentische Ausbildung im Fach Rechtsmedizin in Deutschland: Prüfungen und Evaluation. Rechtsmedizin 31:438–443 33612976 10.1007/s00194-021-00454-z PMC7884059

[17] Heide S, Lessig R, Diers V, Rönsch M, Stoevesandt D (2016) Etablierung der Station „Leichenschau“ im SkillsLab und E-Learning. Rechtsmedizin 26:90–96 10.1007/s00194-015-0070-1

[18] Biolik A, Heide S, Lessig R, Hachmann V, Stoevesandt D, Kellner J, Jäschke C, Watzke S (2018) Objective structured clinical examination “Death Certificate” station - Computer-based versus conventional exam format. J Forensic Leg Med 55:33–38 29459096 10.1016/j.jflm.2018.02.010

[19] Stoevesandt D, Woydt L, Steglich J et al (2023) Simulation im Wahlfach Rechtsmedizin im Dritten Abschnitt der Ärztlichen Prüfung. Rechtsmedizin 33:59–62 35873499 10.1007/s00194-022-00586-w PMC9295882

[20] Flössel U, Clas S, Willemer M, Sommer M, Poweleit G, Schulze R, Heide S, Erfurt C (2021) Using simulation mannequins and actors in training for external post-mortem examinations -experiences from use in medical students and police officers. J Forensic Leg Med 77:102102 33341020 10.1016/j.jflm.2020.102102

[21] Bossard C, Kermarrec G, Buche C, Tisseau J (2008) Transfer of learning in virtual environments: A new challenge? Virtual Reality 12:151–161 10.1007/s10055-008-0093-y

[22] Richter C, Hoyer S, Lessig R, Stoevesandt D, Schwarz K, Biolik A, Heide S (2020) Aktuelle Trends im Leichenschautraining bei Medizinstudierenden. Braucht man noch eine echte Leiche? Rechtsmedizin 30:318–324 10.1007/s00194-020-00400-5

[23] Siol AF, Peter L-M, Richter C, Lessig R, Junghänel M, Stoevesandt D (2021) Leichenfund – Lehrmodul in der virtuellen Realität. Ärzteblatt Sachsen-Anhalt 32(1/2):41–45

[24] Wissenschaftsrat (2018) Neustrukturierung des Medizinstudiums und Änderung der Approbationsordnung für Ärzte | Empfehlungen der Expertenkommission zum Masterplan Medizinstudium 2020, Wissenschaftsrat. https://www.wissenschaftsrat.de/download/archiv/7271-18.html. Accessed 30 Jan 2024

[25] Bibrack E, Horneff H, Krumm K, Hinrichs J, Mette M (2022) Transferring interprofessional education concepts across sites – experiences and recommendations for practice. GMS J Med Educ 39(1):Doc8 35368841 10.3205/zma001529 PMC8953188

[26] Madea B (2009) Strukturelle Probleme bei der Leichenschau. Rechtsmedizin 19:399–406 10.1007/s00194-009-0638-8

[27] Campobasso CP, Laviola D, Grattagliano I, Strada L, Dell’Erba AS (2015) Undetected patricide: Inaccuracy of cause of death determination without an autopsy. J Forensic Leg Med 34:67–72 26165662 10.1016/j.jflm.2015.05.008

[28] Gleich S, Viehöver S, Stäbler P et al (2017) Falsch bescheinigter natürlicher Tod nach ärztlicher Leichenschau. Rechtsmedizin 27:2–7 10.1007/s00194-016-0132-z

[29] Ebert LC, Nguyen TT, Breitbeck R et al (2014) The forensic holodeck: an immersive display for forensic crime scene reconstructions. Forensic Sci Med Pathol 10:623–626 25315842 10.1007/s12024-014-9605-0

[30] Koller S, Ebert LC, Martinez RM, Sieberth T (2019) Using virtual reality for forensic examinations of injuries. Forensic Sci Int 295:30–35 30554020 10.1016/j.forsciint.2018.11.006

[31] Hölzer H, Freytag J, Sonntag U (2017) Faculty development for small-group-teaching with simulated patients (SP)—design and evaluation of a competency-based workshop. GMS J Med Educ 34(4):Doc42 29085886 10.3205/zma001119 PMC5654117

[32] Entner C, Fleischmann A (2017) Didaktische Modelle für den Kleingruppenunterricht. Man Med 55:355–359 10.1007/s00337-017-0323-z

[33] Speidel R, Schneider A, Körner J, Grab-Kroll C, Öchsner W (2021) Did video kill the XR star? Digital trends in medical education before and after the COVID-19 outbreak from the perspective of students and lecturers from the faculty of medicine at the University of Ulm. GMS J Med Educ 38(6):Doc101 34651059 10.3205/zma001497 PMC8493844

[34] Kwon C (2019) Verification of the possibility and effectiveness of experiential learning using HMD-based immersive VR technologies. Virt Reality 23(1):101–118 10.1007/s10055-018-0364-1

